# Bovine leukemia virus proviral load is more strongly associated with bovine major histocompatibility complex class II *DRB3* polymorphism than with *DQA1* polymorphism in Holstein cow in Japan

**DOI:** 10.1186/s12977-019-0476-z

**Published:** 2019-05-16

**Authors:** Shin-nosuke Takeshima, Ayumu Ohno, Yoko Aida

**Affiliations:** 1 Viral Infectious Diseases Unit, RIKEN, Wako, Saitama 351-0198 Japan; 2 Photonics Control Technology Team, RIKEN Center for Advanced Photonics, Wako, Saitama 3510198 Japan; 3 Nakamura Laboratory, Baton Zone Program, RIKEN Cluster for Science, Technology and Innovation Hub, Wako, Saitama 351-0198 Japan; 4 Department of Food and Nutrition, Jumonji University, Niiza, Saitama 352-8510 Japan

**Keywords:** *BoLA*-*DRB3*, *BoLA*-*DQA1*, Bovine leukemia virus, Proviral load, Japanese Holstein

## Abstract

Bovine leukemia virus (BLV) causes enzootic bovine leukosis and is closely related to the human T-lymphotropic virus. Bovine major histocompatibility complex (BoLAs) are used extensively as markers of disease and immunological traits in cattle. For BLV diagnosis, proviral load is a major diagnosis index for the determination of disease progression and transmission risk. Therefore, we investigated the frequency of *BoLA*-*DRB3* alleles, *BoLA*-*DQA1* alleles, and haplotypes of *BoLA class II* isolated from the heads of 910 BLV-infected cows out of 1290 cows assessed from BLV-positive farms, in a nationwide survey from 2011 to 2014 in Japan. Our aim was to identify *BoLA* class II polymorphisms associated with the BLV proviral load in the Holstein cow. The study examined 569 cows with a high proviral load and 341 cows with a low proviral load. Using the highest odds ratio (OR) as a comparison index, we confirmed that *BoLA*-*DRB3* was the best marker for determining which cow spread the BLV (OR 13.9 for *BoLA*-*DRB3*, OR 11.5 for *BoLA*-*DQA1*, and OR 6.2 for *BoLA* class II haplotype). In addition, DRB3*002:01, *009:02, *012:01, *014:01, and *015:01 were determined as BLV provirus associated alleles. *BoLA*-DRB3*002:01, *009:02, and *014:01 were determined as resistant alleles (OR > 1), and *BoLA*-DRB3*012:01 and *015:01 were determined as susceptible alleles (OR < 1). In this study, we showed that *BoLA*-*DRB3* was a good marker for determining which cow spread BLV, and we found not only one resistant allele (*BoLA*-DRB3*009:02), but also two other disease-resistant alleles and two disease-susceptible alleles. This designation of major alleles as markers of susceptibility or resistance can allow the determination of the susceptibility or resistance of most cows to disease. Overall, the results of this study may be useful in eliminating BLV from farms without having to separate cows into several cowsheds.

Bovine leukemia virus (BLV), which is the causative agent of enzootic bovine leukosis (EBL), belongs to the family Retroviridae (genus *Deltaretrovirus*), together with human T-lymphotropic virus types 1 and 2 (HTLV-1 and -2) [1]. At present, BLV is widely distributed in cow populations [2–7]. The virus was identified in 1969 as an infectious retrovirus, and it induces CD5^+^B- cell leukemia/lymphoma in 1% to 5% of infected cows of 5 to 10 years of age [1]. Therefore, the virus was categorized as a non-severe infectious disease in several regions, including USA, South America, and some Asian countries [8]. This decision has resulted in the number of BLV-infected cows increasing in these areas; for example, in Japan, almost 40% of cows are infected [9], and in the USA, 80% of farms have become BLV-positive [8]. At present, BLV elimination is quite difficult, due to its high infection rate and the existence of heavily infected cows. Moreover, recently emerged BLV infections can cause earlier EBL onset, high accident rates, and low rates of conception and low milk production [6, 10–15]. Therefore, techniques for identifying high-risk cows are urgently needed to mitigate economic losses. It has been posited that cows classified as high-risk show a proviral load of over 14,000 copies/10^5^ cells and 18,000 copies/10^5^ cells in blood samples secreting BLV provirus into nasal and saliva, respectively [16]. It has been suggested that these cows may cause a high-risk for BLV transmission via coming into direct contact with healthy cows. In addition, it appears that proviral load correlates not only with BLV infection, but also with BLV disease progression [17–19]. Thus, BLV proviral load is an important index for estimating the stage of BLV infection.

Studies on BLV-associated host factors identified polymorphisms within the bovine major histocompatibility complex (MHC) (BoLA) [20–29]. BoLA is a highly polymorphic and tightly linked gene cluster [30]. Functionally, the *BoLA* class II gene is classified into two groups, DR and DQ. The DR molecule was constructed from a single *DRA* locus and a single *DRB3* locus, and DQ molecules were constructed from at least two *DQA* loci and two *DQB* loci [31]. To date, 136 *DRB3*, 65 *DQA*, and 87 *DQB* alleles have been registered on the IPD-MHC database (http://www.ebi.ac.uk/ipd/mhc/bola). Recently BLV proviral load quantification methods have been developed [17, 32] and several studies have successfully identified SNPs or *BoLA*-*DRB3* alleles that are associated with increasing or suppressing the BLV provirus load [27–29, 33–36]. However, the results of association studies that compared the frequencies of *BoLA* alleles in low proviral load cows with those in high proviral load cows were strongly affected by the allele frequencies in normal cows, and there is little information on how the allele frequencies were stable year-on-year in a countries.

For BLV diagnosis, proviral load is one of the major diagnosis indices for determining disease progression and transmission risk. Therefore, in this study, we investigated *BoLA*-*DRB3* and *BoLA*-*DQA1* allele frequencies in Japan over 4 years, performed an association study using the specific *BoLA* class II allele to determine BLV provirus load in Holstein cows, and determined proviral load-associated polymorphisms using cow which collected among 4 years.

We collected blood samples from 1290 cow heads over 6 months old from BLV-positive farms in a nationwide survey in Japan from 2011 to 2014, isolated genomic DNA and sera from peripheral blood. Cows determined as BLV-positive by anti-BLV gp51 antibody ELISA kit using sera (JNC Corporation, Kanagwa Japan) (Table 1) and the BLV proviral load measured by the BLV-CoCoMo-qPCR-2 method [32] using genomic DNA. First, we confirmed the allele frequencies of *BoLA*-*DRB3* gene in each 4 years and confirmed the allele frequency is stable in these 4 years in Holstein in Japan (Fig. 1). Next, our previous report showed that cows with a detected proviral load of over 14,000 copies/10^5^ cells (as determined by the BLV-CoCoMo-qPCR-2 method) secreted BLV provirus into nasal secretions [16]. Thus, these cows may be high-risk transmitters. Therefore, we here categorized the 910 BLV-infected cows into two groups, as follows: (i) cows with proviral load over 10,000 copies/10^5^ cells—high-risk BLV spreader cows, and (ii) cows with proviral load under 10,000 copies/10^5^ cells—low-risk BLV spreader cows (Table 1). The 910 cow heads tested were separated into 341 heads of “low-risk spreaders” and 569 heads of “high-risk spreaders.”

**Table 1:** Samples collected from Japanese BLV-positive farms (over 60% positive rate in the test prior to collection for this study) and distribution of proviral load in BLV-positive cow

	Year				Total
	2011	2012	2013	2014	
Number of animals					
Collected	322	390	290	288	1290
BLV-positive	222	275	199	214	910
High risk spreader*	137	160	133	139	569
Low risk spreader**	85	115	66	75	341
Distribution of age in BLV positive cow (month)					
Average of age	57.1	58.0	56.0	55.3	56.7
Maximum age	148	191	142	134	191
Minimum age	12	11	15	17	11
Standard deviation of age	24.2	28.6	24.7	22.2	25.3
Distribution of proviral load in BLV positive cow (copies per 10^5^ cells)					
Average	33564	27,927	42,850	42,014	35,878
Maximum	137,905	135,662	154,306	135,950	154,306
Minimum	0	0	0	0	0
Standard deviation	32,786	28,945	38,382	39,536	35,285

* High risk spreader; proviral load > 10,000/10^5^ cells

** Low risk spreader; proviral load ≤ 10,000/10^5^ cells

**Fig. 1:**
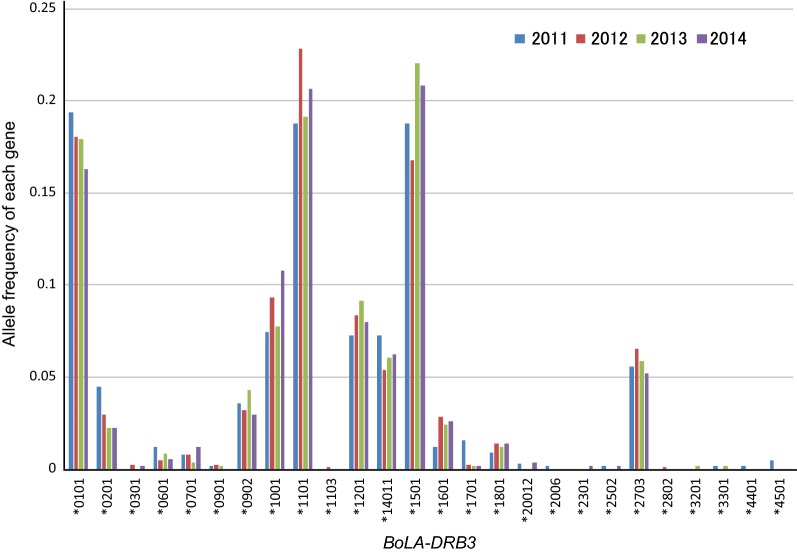
Transition of *BoLA*-*DRB3* allele frequencies of Holstein cow in Japan from 2011 to 2014. DNA samples were collected from the blood of 1290 Holstein cows belonging to BLV-positive commercial dairy farms located in the 23 prefectures of Japan, from 2011 to 2014 by Ohno et al. [18] and genotyped for *BoLA*-*DRB3* alleles with PCR-sequence based typing (SBT) developed previously [37]. In total, 644 alleles were detected at 322 cows in 2011, 780 alleles at 390 cow in 2012, 580 alleles at 290 cow in 2013 and 576 alleles at 288 cow in 2014

Next, these 910 cows were subjected to *BoLA*-*DRB3* genotyping using a PCR-sequence-based typing (PCR-SBT) method [37]. From 910 BLV-positive cows, a total of 1820 *BoLA*-*DRB3* alleles were detected, which were classified into 23 types of known *BoLA*-*DRB3* alleles (Fig. 2). *BoLA*-*DRB3* allele frequencies of these two groups, i.e., 682 alleles originating from low-risk spreaders and 1138 alleles originating from high-risk spreaders, were calculated, and estimated *p* values and odds ratios (ORs) for each *BoLA*-*DRB3* allele in the two spreader groups were compared (Fig. 2). If the allele which significantly low frequency in low risk spreader than high risk spreader (OR > 1), we determined that the allele was resistant allele. Moreover, in the case that the allele which significantly high frequency in high risk spreader than low risk spreader (OR > 1), the allele was determined as susceptible allele. From these 23 *BoLA*-*DRB3* alleles, DRB3*002:01, DRB3*009:02, DRB3*012:01, DRB3*014:01:01, and DRB3*015:01 were determined as BLV provirus-associated alleles. *BoLA*-DRB3*002:01, DRB3*009:02, and DRB3*014:01:01 were determined to be alleles associated with BLV resistance (OR > 1), whereas *BoLA*-DRB3*012:01 and DRB3*015:01 were determined to be alleles associated with BLV susceptibility (OR < 1).

**Fig. 2:**
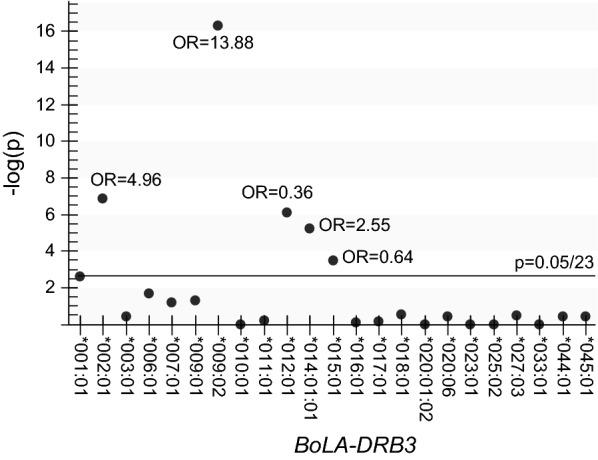
Association study to determine the significance of the detection frequency of the *BoLA*-*DRB3* allele compared with those of 682 alleles from low-risk spreaders and 1138 alleles from high-risk spreaders.. BLV-positive cows were diagnosed using an anti-BLV antibody ELISA Kit (JNC, Tokyo, Japan), which is used to detect anti-Env gp51 antibodies from serum samples, according to the manufacturer’s instructions. BLV-positive cows were subjected to BLV proviral load calculation using the BLV-CoCoMo-qPCR-2 system (RIKEN Genesis, Kanagawa, Japan) [32]. The 910 BLV-infected cows were classified into two groups: (i) cows with proviral load over 10,000 as high-risk BLV spreader cows (N = 569), and (ii) cows with proviral load under 10,000 as low-risk BLV spreader cows (N = 341). Fisher’s exact test was performed for estimating *p* values and odds ratios (ORs) were used for detecting the association of each BoLA-DRB3 allele and BLV proviral load, using R version 3.4.2 statistical analysis software [43]. The X-axis shows the allele number for *BoLA*-*DRB3* and the Y-axis shows the minus multiplied common logarithm for *p* values. The alleles with *p* values < 0.00217 (0.05/23 alleles, the statistical significance threshold) were determined to be resistant when OR > 1 and susceptible when OR < 1

There are *DR* and *DQ* genes embedded in the *BoLA class II* region, and these genes were closely linked to each other [30]. Indeed, we previously identified 39 *DRB3*-*DQA1* haplotypes in 507 Japanese Black cows [38]. Therefore, to determine the effect of other class II genes, we genotyped the second polymorphic class II genes, such as the *DQA1* gene. The 910 Japanese Holstein cow heads were subjected to genotyping of the *BoLA*-*DQA1* gene using a PCR-SBT method [39] and 899 cows were succeeded to genotyping for *BoLA*-*DQA1* alleles. These 899 cows were divided into low-risk (N = 336) and high-risk spreaders (N = 563), based on whether their proviral load was under or over 10,000 copies/10^5^ cells, respectively. In total, 1798 *BoLA*-*DQA1* alleles were detected, and these alleles were assigned as one of 14 kinds of known *BoLA*-*DQA1* alleles (Fig. 3). *BoLA*-*DQA1* allele frequencies of these two groups (672 alleles originating from low-risk spreaders and 1126 alleles originating from high-risk spreaders), were analyzed using Fisher’s exact test. Three kinds of B*oLA*-*DQA1* allele—DQA1*002:04, DQA1*012:01:01, and DQA1*014:02—were significantly associated with the high proviral load (Fig. 3). DQA1*002:04 and DQA1*014:02 showed ORs > 1 (12.8 and 2.47, respectively), as these two alleles were disease resistant. Conversely, the OR of DQA1*012:01 was 0.34 and the allele indicated disease susceptibility.

**Fig. 3:**
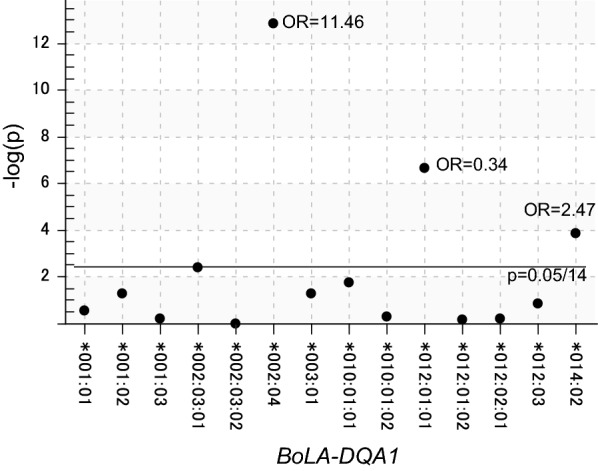
Association study to determine the significance of detection frequency of the *BoLA*-*DQA1* allele compared with 672 alleles from low-risk spreaders and 1126 alleles from high-risk spreaders. The same samples as described in Fig. 1 were genotyped for *BoLA*-*DQA1* alleles with PCR-sequence based typing (SBT) developed previously [39]. Fisher’s exact test was performed for estimating *p* values and odds ratios (ORs) were used to detect the association of *BoLA*-*DQA1* alleles with BLV proviral load. The X-axis shows the allele number for *BoLA*-*DQA* and Y-axis shows the minus multiplied common logarithm for *p* values. The alleles with *p* values < 0.00357 (0.05/14 alleles) were determined as resistant when OR > 1 and susceptible when OR < 1

Notably, *DRB3* and *DQA1* were highly linked [38]: for example, DRB3*009:02 was linked with DQA1*002:04 and DRB3*014:01:01 was linked with DQA1*014:02 in Japanese Holstein cows [35]. Therefore, we identified that DRB3*009:02-DQA1*002:04 and DRB3*014:01:01-DQA1*014:02 haplotypes were indicated disease resistance. Table 2 shows that animals with the resistant haplotype were detected at a significantly higher level in the low proviral load group compared with the high proviral load group. However, the OR was lower when the *DRB3*-*DQA1* haplotype was used as a marker (OR 6.16) than when the *DRB3* allele alone was used (OR 13.88).

**Table 2:** Association between cows with BLV-resistant *DRB3*-*DQA1* haplotypes and cows without BLV-resistant *DRB3*-*DQA1* haplotypes (*p* value = 9.029 × 10^−15^, odds ratio = 6.168086)

		Category of proviral load	
		Low (≤ 10,000 copies/10^5^ cells)	High (> 10,000 copies/10^5^ cells)
Number of *DRB3*-*DQA1* haplotype	BLV resistant*	544	1192
	Non BLV resistant	62	22

* BLV resistant haplotype; *BoLA*-DRB3*009:02-DQA1*002:04 or *BoLA*-DRB3*014:01:01-DQA1*014:02

In this study, we used three markers, *BoLA*-*DRB3*, *BoLA*-*DQA1*, and *BoLA class II* haplotypes, to determine the risk of BLV spread through cows in the farm environment. Using the biggest OR as a comparison index, we confirmed that *BoLA*-*DRB3* was the best marker for determining which cow spread the BLV (OR 13.9 for *BoLA*-*DRB3*, OR 11.5 for *BoLA*-*DQA1*, and OR 6.2 for *BoLA class II* haplotype).

The most strongly associated allele was *BoLA*-DRB3*009:02, which was determined to be a BLV-resistant allele in our study, and was also detected in several studies, such as those by Julliarena et al. [36], Miyasaka et al [35], Forletti et al [40], Lutzelscheab et al. [21], Carignano et al [34], and Hayashi et al [33]. Moreover, we explored the other resistant alleles, *BoLA*-DRB3*002:01 and DRB3*014:01:01, and susceptible alleles, DRB3*012:01 and DRB3*015:01. The effects of these alleles were weaker than that of *BoLA*-DRB3*009:02, but they were more frequently detected in the farm [41]. Therefore, obtaining information about these common alleles is more important than obtaining information about rare alleles. It is true that the PVL we determined is only about single time point, the PVL may be changing in future. However, in our limited data in lab, the PVL tends to be stable at least 6 months. Needs more research to confirm how long the PVL shows stable. As the BLV PVL is the most variable quantitative index for assessing the risk of BLV transmission [42], the information about disease susceptible and resistant alleles may be useful to eliminate BLV from the farm without separating cows into several sheds.

## Data Availability

All data generated or analyzed during this study are included in this published article and its additional file.

## Abbreviations

BLV: bovine leukemia virus
BoLA: bovine leukocyte antigen
EBL: enzootic bovine leukosis
HTLV: human T-lymphotropic virus
MHC: major histocompatibility complex
OR: odds ratio
PCR-SBT: PCR-sequence based typing
PVL: proviral load
